# Relationship between circulating tumor cells and epithelial to mesenchymal transition in early breast cancer

**DOI:** 10.1186/s12885-015-1548-7

**Published:** 2015-07-22

**Authors:** M. Mego, Z. Cierna, P. Janega, M. Karaba, G. Minarik, J. Benca, T. Sedlácková, G. Sieberova, P. Gronesova, D. Manasova, D. Pindak, J. Sufliarsky, L. Danihel, JM Reuben, J. Mardiak

**Affiliations:** 1 2nd Department of Oncology, Faculty of Medicine, Comenius University and National Cancer Institute, Klenova 1, 833 10 Bratislava, Slovak Republic; 2 Translational Research Unit, Bratislava, Slovakia; 3 Department of Pathology, Bratislava, Slovakia; 4 Institute of Molecular Biomedicine, Faculty of Medicine, Comenius University, Bratislava, Slovakia; 5 National Cancer Institute, Bratislava, Slovakia; 6 Institute of Normal and Pathological Physiology, Bratislava, Slovakia; 7 Cancer Research Institute, Slovak Academy of Sciences, Bratislava, Slovakia; 8 Slovak Medical University, Bratislava, Slovakia; 9 Department of Hematopathology, The University of Texas MD Anderson Cancer Center, Houston, TX USA

**Keywords:** Circulating tumor cells, Epithelial-to-mesenchymal transition, Early breast cancer

## Abstract

**Background:**

Circulating tumor cells (CTCs) play a crucial role in tumor dissemination and are an independent survival predictor in breast cancer (BC) patients. Epithelial to mesenchymal transition (EMT) is involved in cancer invasion and metastasis. The aim of this study was to assess correlation between CTCs and expression of EMT transcription factors TWIST1 and SLUG in breast tumor tissue.

**Methods:**

This study included 102 early BC patients treated by primary surgery. Peripheral blood mononuclear cells (PBMC) were depleted of hematopoietic cells using RossetteSep™ negative selection kit. RNA extracted from CD45-depleted PBMC was interrogated for expression of EMT (TWIST1, SNAIL1, SLUG, FOXC2 and ZEB1) and epithelial (KRT19) gene transcripts by qRT-PCR. Expression of TWIST1 and SLUG in surgical specimens was evaluated by immunohistochemistry and quantified by multiplicative score.

**Results:**

CTCs were detected in 24.5 % patients. CTCs exhibiting only epithelial markers were present in 8.8 % patients, whereas CTCs with only EMT markers were observed in 12.8 % of pts and CTCs co-expressing both markers were detected in 2.9 % pts. We observed lack of correlation between CTCs and expression of TWIST1 and SLUG in breast cancer cells or cancer associated stroma. Lack of correlation was observed for epithelial CTCs as well as for CTCs with EMT.

**Conclusions:**

In this translational study, we showed a lack of association between CTCs and expression of EMT-inducing transcription factors, TWIST1 and SLUG, in breast tumor tissue. Despite the fact that EMT is involved in cancer invasion and metastasis our results suggest, that expression of EMT proteins in unselected tumor tissue is not surrogate marker of CTCs with either mesenchymal or epithelial features.

## Background

Circulating tumor cells (CTCs) have a crucial role in the metastatic cascade, tumor dissemination and progression. Prognostic value of CTCs was demonstrated by numerous trials for metastatic as well as primary breast cancer [1–4]. However, CTCs represent a heterogeneous population of cells with different phenotypes and biological value [5].

To successfully execute the metastatic cascade, epithelial tumor cells must detach from the primary tumor, pass through the peripheral circulation, extravasate at the distant site and establish a new tumor. Increased number of studies demonstrated that cancer cells often undergo epithelial to mesenchymal transition (EMT), to acquire the traits needed to execute the multiple steps of metastasis [6]. During the EMT, epithelial cells downregulate epithelial-related genes, acquire mesenchymal gene expression and undergo major changes in their cytoskeleton that result in loss of cell–cell contacts and cell polarity leading to increased motility and invasiveness [7]. EMT is associated with de novo expression of stem cell markers and acquisition of functional stem cell properties [8]. Several signaling pathways are involved in the induction of EMT including TGF-β1, Wnt, Notch or Hedgehog signaling. Overexpression of transcription factors (TF), including TWIST1, SNAIL1, SLUG, ZEB1 and/or FOXC1,2, can induce EMT in mammary epithelial cells and/or breast cancer cells as well [9, 10].

Several studies evaluated expression of EMT transcription factors (SNAIL, SLUG, ZEB1, ZEB2, TWIST1, and TWIST2) in breast cancer tissue sections [11–14]. Studies showed that the level of SNAIL and TWIST expression was associated with a worse patient outcome [15–17]. Moreover, some types of breast cancer including basal like, claudin low and metaplastic carcinoma show upregulation of mesenchymal markers and reduced levels of epithelial markers, consistent with EMT [18–20].

Experimental and clinical data suggest that the EMT has an important role in the generation of CTCs and the acquisition of resistance to therapy. Inhibition of TWIST in the highly metastatic 4 T1 murine mammary cell line reduced both metastatic burden and the number of CTCs in mice bearing xenograft mammary tumors, thus linking EMT, metastasis and the presence of CTCs [10]. Experimental and translational research data suggest that there is a continuum of development of CTCs that range from one end of the spectrum (epithelial phenotype) to the other end of the spectrum (mesenchymal phenotype) and include those with a partial EMT phenotype [5, 21–23]. Recently, it was showed that CTCs exhibit dynamic changes in epithelial and mesenchymal composition [21].

In this translational study, we hypothesized, that activation of EMT in primary tumor could be involved in CTCs release into peripheral blood (PB) and therefore CTCs will be detected more often in breast cancer patients with high expression of EMT-induced TFs in primary tumor or tumor associated stroma. Therefore, we examined expression of EMT induced TFs on breast tumor tissue as well as tumor associated stroma and correlated them with CTCs in peripheral blood. We elected to study the early breast cancer model, to avoid the factor of metastatic sites heterogeneity on analyzed variables.

## Methods

### Study patients

As a part of ongoing translational study (Protocol TRU-SK 002; Chair: M. Mego), 102 patients with stages I–III primary breast cancer (PBC) who were undergoing definitive surgery were included. From each patient we obtained peripheral blood for CTCs detection and corresponding paraffin-embedded tumor tissue. The blood was drawn in the morning on the day of surgery, before surgical procedure. Each patient was given a complete diagnostic evaluation to exclude the presence of distant metastasis. Patients with concurrent malignancy other than non-melanoma skin cancer in the previous 5 years were excluded as well. In all patients, data regarding age, tumor stage, histology, regional lymph node involvement, hormone receptor status, and HER2 status were also recorded.

The study was approved by the Institutional Review Board (IRB) of the National Cancer Institute of Slovakia and was conducted between March and December 2012 and each participant signed informed consent before study enrollment. Healthy donors (*N* = 60) were age-matched women without breast cancer who were recruited and consented according to the IRB-approved protocol. Each participant signed informed consent.

### Detection of CTC in peripheral blood

CTC were detected in peripheral blood by quantitative real-time polymerase chain reaction (qRT-PCR) based assay utilizing CD45 positive (CD45+) cells depletion for CTCs enrichment, as described previously [24, 25].

#### RNA extraction and cell lines

Peripheral blood was subjected to CD45 depletion using RossetteSep™ kit (StemCell technologies) according to the manufacturer’s instructions. CD45-depleted cells were mixed with 500 μl of TRIzol® LS Reagent (Invitrogen Corporation, Carlsbad, CA) and stored at −80 °C until it was necessary to extract RNA according to the manufacturer’s instructions. The precipitated pellet containing RNA was dissolved in 50 μl of nuclease-free water. All RNA preparation and handling steps took place in a laminar flow hood, under RNase-free conditions. RNA concentration was determined by absorbance readings at 260 nm (median = 5.95 ng/μl, range: 1.7 – 38.3). RNA extracted from HeLa, HCT 116, MCF7 and MDA-MB-231 cells were used as positive controls.

#### Identification of gene transcripts in CD45-depleted subsets

Isolated RNA was subjected to quantitative RT-PCR (qRT-PCR) to detect EMT-inducing TF gene transcripts (TWIST, SNAIL1, SLUG, ZEB1 and FOXC2) and epithelial antigen (KRT19). In brief, 2.5 μL of RNA were placed in 25 μL of reaction volume containing 12.5 μL of QuantiFast Probe RT-PCR Kit Master Mix, 0.25 μL QuantiFast RT Mix, 8.5 μL water and 1.25 μL of primers. The following TaqMan assays were purchased from LifeTechnologies (USA): TWIST1: Hs00361186_m1; SNAIL1: Hs00195591_m1; SLUG: Hs00161904_m1; ZEB1: Hs01566408_m1; FOXC2: Hs01013460_s1; GAPDH: Hs99999905_m1; KRT19 Hs00761767_s1. Amplicons or probes spanned intron–exon boundaries, with the exception of FOXC2 and KRT19. Amplification was performed on an Eppendorf Realplex Real-Time PCR system (Eppendorf, Germany) using the cycling program: 95 °C for 10 min; 40 cycles of 95 °C for 15 s and 60 °C for 60 s. All samples were analyzed in triplicate. Calibrator samples were run with every plate to ensure consistency of the PCR. For all fluorescence-based RT-PCR, fluorescence was detected between 0 and 40 cycles for the control and marker genes in single-plex reactions, which allowed for the deduction of the cycles at threshold (Ct) value for each product. Expression of the genes of interest was calibrated against expression of the housekeeping gene, GAPDH. Target cDNA was quantified using the delta-Ct method with the formula: 1 = 2 Ct(target-GAPDH).

#### CTC definition

Patient samples with higher KRT19 gene transcripts than those of healthy donors were scored as epithelial CTCs positive (CTC_EP), while patient samples with higher EMT-TF (TWIST1, SNAIL1, SLUG, ZEB1 and FOXC2) gene transcripts than those of healthy donors were scored as CTC_EMT positive. Expression of at least one of the markers (either epithelial or mesenchymal) at levels above the defined cutoff was sufficient to define a sample as CTC positive.

The highest expression levels of the KRT19 and EMT-inducing TF gene transcripts relative to that of GAPDH were 3.4 × 10 ^−3^ (median 2.8 ×10^−6^, range: 0–3.4 × 10^−3^) for KRT19, 7.5 × 10^−4^ (median 0, range: 0–7.5 × 10^−4^) for TWIST1, 3.8 × 10^−2^ (median 0.003135, range: 5.0 × 10^−4^ - 3.8 × 10^−2^) for SNAIL1, 1.7 × 10^−1^ (median 1.4 × 10^−2^, range: 2.2 × 10^−3^ – 1.7 × 10^−1^) for ZEB1 and 4.0 × 10^−2^ (median 4.0 × 10^−3^, range: 1.7 × 10^−4^ – 4.0 × 10^−2^) for FOXC2, while SLUG transcripts were not detected in any of the samples from healthy donor. These highest expression values in healthy donors were used as “cutoff” to determine CTCs positivity.

### Tumor pathology

Pathology review was conducted at the Department of Pathology, Faculty of Medicine, Comenius University, by two pathologists (ZC and PJ) associated with the study.

### Diagnosis and tumor samples

The study included tumor specimens from 102 patients. All specimens were classified according to the WHO Classification of 2004. The block containing the most representative part of the tumor was identified by H&E microscopy and used for IHC analysis.

### Tissue microarray construction

According to tumor histology, one or two representative tumor areas were identified on H&E sections. Sections were matched to their corresponding wax blocks (the donor blocks), and 3-mm diameter cores of the tumor were removed from these donor blocks with the multipurpose sampling tool Harris Uni-Core (Sigma-Aldrich, Steinheim, Germany) and inserted into the recipient master block. The recipient block was cut into 5-μm sections, and the sections were transferred to coated slides.

### Immunohistochemical (IHC) staining

Slides were deparaffinised and rehydrated in phosphate buffered saline solution (10 mM, pH 7.2). The tissue epitopes were demasked using the automated water bath heating process in Dako PT Link (Dako, Glostrup, Denmark); the slides were incubated in TRIS-EDTA retrieval solution (10 mM TRIS, 1 mM EDTA pH 9.0) at 98 °C for 40 min (TWIST1 staining) or for 20 min (SLUG staining). The slides were subsequently incubated overnight at room temperature with the primary mouse monoclonal antibody against TWIST1 (Abcam, Twist2C1a, ab50887) diluted 1:100; or overnight at 4 °C with the primary mouse monoclonal antibody against SLUG (Santa Cruz, A-7, sc-166476) diluted 1:50 in Dako REAL antibody diluent (Dako, Glostrup, Denmark) and immunostained using rabbit anti-mouse immuno-peroxidase polymer (EnVision FLEX/HRP, Dako, Glostrup, Denmark) for 30 min at room temperature, according to the manufacturer’s instructions. For visualisation, the diaminobenzidine substrate-chromogen solution was used (DAB, Dako, Glostrup, Denmark) for 5 min. Finally, the slides were counterstained with haematoxylin. As tumour associated stroma, the stromal cells between tumour nests, adjacent to tumour cells were evaluated. Cancer associated stroma was indicated by vimentin-positive (Dako, Monoclonal mouse anti-vimentin clone V9, code IR630) and pan-cytokeratin-negative (Dako, Monoclonal mouse anti-human clones AE1/AE3, code M3515). Samples of breast carcinoma with high expression of TWIST1 served as the positive control TWIST1 as described previously [26] and placental tissue served as a positive control for SLUG. As negative control, breast tissue was subjected to the same procedure without staining with the primary antibody.

### Immunohistochemical stain scoring

Tumor cores were independently assessed by two pathologists (ZC and PJ) who were blinded to clinico-pathological data. In cases of disagreement, the result was reached by consensus. The result of the IHC analyses was expressed by a weighted histoscore, evaluating both the percentage of positive cells (PP) and the staining intensity (SI) of the nuclei. Briefly, the proportion of cells with nuclear staining was multiplied by the intensity of staining to provide a score ranging from 0–300. The score was calculated as follows: Score = (0 × percentage not stained) + (1 × percentage weakly stained) + (2 × percentage moderately stained) + (3 × percentage strongly stained) [15].

### Statistical analysis

Patient characteristics were tabulated. The patients’ characteristics were summarized using the median (range) for continuous variables and frequency (percentage) for categorical variables. Normality of distribution was tested by the Kolmogorov-Smirnoff test. If normally distributed, sample means were tested by Student *t*-test or analysis of variance (ANOVA) with Bonferroni’s or Tamhane’s corrections, depending on homogeneity of variance. Nonparametric Mann–Whitney *U* or Kruskal-Wallis H test were used for non-normally distributed data. Pearson’s or Spearman’s correlations were used according to the normality of data. All p values presented are two-sided, and associations were considered significant if the p value is less or equal to 0.05. Statistical analyses were performed using NCSS 2007 software (Hintze J, 2007, Kaysville, Utah, USA).

## Results

The study population consisted of 102 primary breast cancer patients with median age of 60 years (range: 37–83 years). Patients’ characteristics are shown in Table 1. There were 86 (84.3 %) patients with estrogen receptor positive (ER) and/or progesterone receptor positive (PR) tumors; 16 (15.7 %) patients with HER-2/neu amplified tumors.

**Table 1:** Patients characteristics

Variable	Number	Percent
All	102	100
T-stage		
1	64	62.7
>1	38	37.3
N-stage		
0	60	58.8
>1	42	41.2
Grade		
1 and 2	60	58.8
3	40	39.2
Histology		
Invasive ductal carcinoma	85	83.3
Other	17	16.7
Hormone receptor status		
Negative for both	16	15.7
Positive for either	86	84.3
HER2 status		
Positive	16	15.7
Negative	86	84.3
Ki 67 (cut-off 14 %)		
Low	51	50.0
High	51	50.0
Epithelial CTC		
Present	12	11.8
Absent	90	88.2
EMT CTC		
Present	16	15.7
Absent	86	84.3
Any CTC		
Present	25	24.5
Absent	77	75.5

### CTC detection

To determine overexpression of the EMT-inducing TF gene transcripts and KRT19 in PBC patients, we compared the expression levels in patient samples with those of HDs. Totally, CTCs were detected in 25 (24.5 %) of patients. CTCs with only epithelial markers were present in peripheral blood of 9 (8.8 %) patients; CTC with EMT only phenotype were present in 13 (12.8 %) of patients; in 3 (2.9 %) of patients CTCs exhibited both epithelial and mesenchymal markers (Table 2). In one patient sample, there was overexpression of two EMT-inducing TF gene transcripts (SLUG and TWIST1), e.g., expression of both genes were higher than the cut-off value in the same sample.

**Table 2:** CTC detection and expression of the genes in CD45 depleted peripheral blood at levels higher than those of healthy donors

Gene	Number of positive samples	% of positive samples
KRT19	12	11.8
TWIST1^a^	2	2.0
SNAIL1	0	0.0
SLUG^a^	13	12.8
ZEB1	0	0.0
FOXC2	2	2.0
CTC_EP only	9	8.8
CTC_EMT only	13	12.8
CTC co-expressing both markers	3	2.9
Any CTC	25	24.5

^a^In one patient sample, there was overexpression of two EMT-inducing TF gene transcripts (SLUG and TWIST1)

### CTCs and EMT- inducing transcription factors in breast cancer cells and tumor associated stroma

**Fig. 1:**
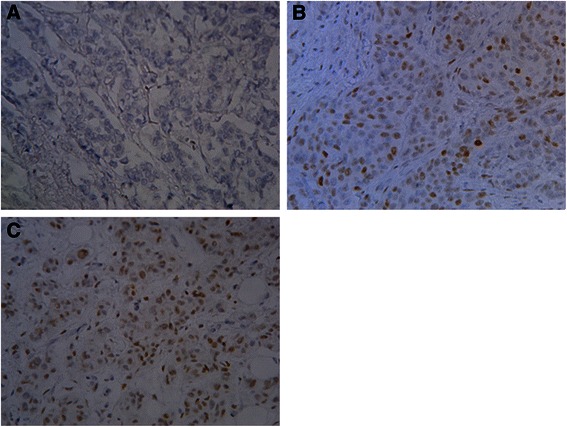
TWIST1 expression in primary breast tumours. Immunohistochemical reaction with anti-TWIST1 monoclonal antibody. Original magnification × 400 visualisation with 3,3’-diaminobenzidine . **a** staining intensity 0, **b** staining intensity 1, **c** staining intensity 2. There were no tumours with staining intensity 3

**Fig. 2:**
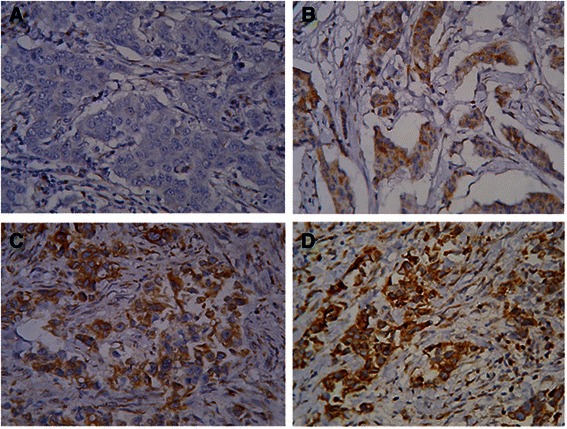
SLUG expression in primary breast tumours. Immunohistochemical reaction with anti SLUG monoclonal antibody. Original magnification × 400 visualisation with 3,3’-diaminobenzidine. **a** staining intensity 0, **b** staining intensity 1, **c** staining intensity 2, **d** staining intensity 3

Tumor expression of TWIST1 and SLUG are associated with poor outcome in breast cancer patients; therefore, we decided to correlate presence of CTCs in peripheral blood with expression of TWIST1 and SLUG in breast cancer cells and cancer associated stroma (Figs. 1 and 2). Expression of TWIST1 and SLUG were detected in 42 (41.7 %) and 76 (74.5 %) of samples, respectively. Mean ± SEM (standard error of mean) for TWIST1 and SLUG expression in breast cancer cells and tumor associated stroma was 8.6 ± 2.2 vs. 54.4 ± 5.1, *p* < 0.0001, and 40.6 ± 4.2 vs. 37.3 ± 2.8, *p* = 0.12, respectively. We observed correlation between TWIST1 and SLUG expression in tumor stroma (Spearman rho’ = 0.37; *p* = 0.0003). Expression of TWIST1 and SLUG in relation to CTCs and various clinicopathological characteristics is shown in Tables 3 and 4.

**Table 3:** TWIST1 expression in tumor cells and tumor stroma

Variable		TWIST1 expression in breast tumor cells^a^			TWIST1 expression in tumor stroma^a^		
	N	Mean	SEM	*p*-value^b^	Mean	SEM	*p*-value^b^
All	102	8.6	2.2	NA	40.6	4.2	NA
T-stage							
1	64	7.7	2.8	0.05	48.7	5.1	0.0007
>1	38	10.1	3.6		26.0	6.8	
N-stage							
0	60	9.5	2.9	0.87	44.3	5.4	0.09
>1	42	7.3	3.4		35.0	6.6	
Grade							
1 and 2	60	10.2	2.9	0.35	38.7	5.6	0.86
3	40	6.6	3.5		43.6	6.7	
Histology							
IDC	85	9.0	2.4	0.25	44.2	4.5	0.06
Other	17	6.8	5.4		22.5	10.1	
Hormone receptor status							
Negative	16	1.8	5.9	0.23	36.8	11.0	0.53
Positive	86	9.7	2.4		41.2	4.6	
HER2 status							
Positive	16	17.8	5.4	0.74	42.5	10.3	0.75
Negative	86	6.8	2.4		40.2	4.6	
Ki 67 (cut-off 14 %)							
Low	51	9.1	3.1	0.83	39.0	5.9	0.60
High	51	8.1	3.2		42.2	6.0	
Epithelial CTC							
Present	12	4.2	6.4	0.96	43.3	11.9	0.75
Absent	90	9.2	2.4		40.2	4.5	
EMT CTC							
Present	16	7.8	5.5	0.62	42.2	10.3	0.98
Absent	86	8.7	2.4		40.3	4.6	
Any CTC							
Present	25	6.2	4.4	0.71	44.6	8.2	0.68
Absent	77	9.4	2.6		39.1	4.9	

^a^Protein expression evaluated semi quantitatively by immunohistochemistry

^b^Nonparametric Mann–Whitney *U* test

**Table 4:** SLUG expression in tumor cells and tumor stroma

Variable		SLUG expression in breast tumor cells^a^			SLUG expression in tumor stroma^a^		
	N	Mean	SEM	*p*-value ^b^	Mean	SEM	*p*-value ^b^
All	102	54.4	5.1	NA	37.3	2.8	NA
T-stage							
1	64	56.7	6.5	0.42	41.3	3.5	0.08
>1	38	50.5	8.3		30.8	4.5	
N-stage							
0	60	61.3	6.6	0.10	38.0	3.7	0.67
>1	42	44.4	7.9		36.3	4.4	
Grade							
1 and 2	60	62.3	6.4	0.007	38.6	3.7	0.58
3	40	39.5	7.7		35.0	4.5	
Histology							
IDC	85	51.3	5.5	0.18	38.9	3.1	0.28
Other	17	70.6	12.7		28.8	7.0	
Hormone receptor status							
Negative	16	53.8	12.8	0.78	31.9	7.1	0.32
Positive	86	54.5	5.6		38.4	3.1	
HER2 status							
Negative	16	45.3	12.8	0.29	39.4	7.1	0.46
Positive	86	56.1	5.6		36.9	3.1	
Ki 67 (cut-off 14 %)							
Low	51	68.2	6.9	0.002	32.9	3.9	0.09
High	51	39.9	7.0		41.8	4.0	
Epithelial CTC							
Present	12	55.8	14.8	0.81	45.8	8.1	0.41
Absent	90	54.2	5.5		36.2	3.0	
EMT CTC							
Present	16	52.5	12.8	0.97	31.3	7.1	0.29
Absent	86	54.7	5.6		38.5	3.1	
Any CTC							
Present	25	55.2	10.2	0.78	37.2	5.7	0.88
Absent	77	54.1	5.9		37.4	3.3	

^a^Protein expression evaluated semi quantitatively by immunohistochemistry

^b^Nonparametric Mann–Whitney *U* test

We observed a lack of association between CTCs and expression of TWIST1 and SLUG in breast cancer cells or cancer associated stroma. Lack of correlation was consistent for epithelial CTCs as well as for CTC_EMT (Table 5). Moreover, there was a trend for decreased expression of SLUG in tumor associated stroma in patients positive for CTCs_EMT (mean ± SEM: 24.3 ± 7.5 vs. 39.4 ± 3.0, *p* = 0.06).

**Table 5:** Correlation between CTCs and expression of TWIST1 and SLUG in primary tumor

	SLUG^a^		TWIST1^a^	
	Cancer	Stroma	Cancer	Stroma
CTC Epithelial^b^				
Spearman rho’	0.03	0.13	−0.07	0.02
*p*-value^c^	0.79	0.21	0.48	0.82
CTC EMT^b^				
Spearman rho’	0.00	−0.09	−0.01	0.02
*p*-value^c^	0.97	0.41	0.89	0.88
CTC Any^b^				
Spearman rho’	0.04	0.02	−0.06	0.06
*p*-value^c^	0.73	0.87	0.55	0.59

^a^Protein expression evaluated semi quantitatively by immunohistochemistry

^b^CTC detected by quantitative RT-PCR

^c^Spearman’s correlation test

TWIST1 expression was increased in breast cancer cells and decreased in tumor associated stroma in patients with > T1 tumors, while SLUG expression was increased in cancer cells of tumors with low and intermediate grade and in tumors with decreased proliferation (low Ki67). There was no association between expression of TWIST1 and SLUG and ER/PR status, HER2/neu amplification or axillary lymph node status (Tables 3 and 4).

## Discussion

In this translational study, we showed lack of association between CTCs and expression of EMT-inducing transcription factors, TWIST1 and SLUG, in primary breast tumor tissue. Moreover, this observation was consistent for both epithelial CTCs and CTCs with EMT phenotype, as well as for TWIST1 and SLUG expression in breast cancer cells and cancer associated stroma.

Several translational studies demonstrated activation of EMT in a subpopulation of CTCs including expression of EMT inducing TFs on CTCs [21–23]. However, in our study there was no correlation even between CTCs_EMT and expression of EMT TFs in tumor tissue. Demonstration of EMT in tissues is limited by the nature of the EMT process: its transient, dynamic, and reversible characteristics [17]. Moreover, there is great variability in evaluating expression of EMT factors between studies. In a study by van Ness et al., high expression of TWIST1 and SNAIL1 was observed in 50 % and 54 % of patients while Soini et al., detected TWIST1 and SNAIL1 expression in 3.6 % and 3.1 % respectively [15, 17]. In our study we detected expression of TWIST1 in 41 % of samples. We evaluated expression of TWIST1 and SLUG in tumor tissue, but not other EMT inducing TFs, therefore, we cannot exclude, that expression of other EMT TFs could be associated with presence of CTCs in peripheral blood. We observed higher SLUG expression in clinically less aggressive tumors (lower grade, lower proliferation) and higher TWIST1 expression was associated with higher tumor stage. These data are consistent with previous observations [15].

We revealed higher expression of TWIST1 in stromal compartment compared to epithelial cells, while there was no difference in SLUG expression between these two compartments. Expression of EMT-inducing TFs in the stromal compartment of breast carcinomas possibly represents two populations of cells; EMT transformed neoplastic cells and stromal fibroblastic cells that undergo activation of EMT induced TFs due to growth factors produced by the tumor [17].

There are several possible explanations for observed data. One of the possibilities is the intratumoral heterogeneity and CTCs, believed to be released only from the tumor edge, may not comprise the heterogeneous tumor population. Examination of the tissue sections from the bulk of tumor mass could miss the small areas of EMT TFs overexpression, as well. Thus, we canot exclude that relationship between CTCs in peripheral blood and tissue expression of EMT TFs is not dose dependent. Another explanation could be related to limited accuracy of immunohistochemistry to quantitate EMT TFs expression compared to more precise methods such as qRT-PCR. The absence of correlation could be also due to post translational modifications that causes that mRNA and protein levels do not always correlated. Presence of CTCs in peripheral blood is a dynamic state, and it is possible, that this is not mirrored by expression of EMT TFs in primary tumor tissue. Different detection methods are capable of detecting different subpopulations of CTCs with different clinical and biological value [5]. All data regarding CTCs, should be therefore interpreted within the context of the detection method used. In our study we detected CTCs by qRT-PCR methods based on expression of KRT19 and EMT-TFs respectively with pre-enrichment step utilizing CD45 negative selection, unfortunately, CD45 depleted cells do not necessarily contain only CTCs. Therefore we defined CTCs positivity based on the cut-off value that was established as the highest expression of corresponding gene in population of healthy donors. However, we cannot exclude correlation between expression of EMT-TF in primary tumor and CTCs detected by different detection method. Finally, limited sample size could affect study results; however, we did not observe nor the trend for correlation between CTCs and expression of EMT TFs in primary tumor. Our data suggest, that EMT TFs expression in unselected tumor tissue did not play a major role in CTCs release, and it is possible, that other factors or signaling pathways are more closely associated with CTCs. Recently, it was identified tumor gene expression profile able to reveal patients with detectable CTCs in primary breast cancer patients [27]. In that study, EMT-TFs TWIST and SLUG were not part of CTC-predictive profile, but definition of CTCs was different compared to our study [27].

## Conclusion

In conclusion, in this prospective translational study, we showed for the first time a lack of association between CTCs in peripheral blood and expression of EMT-inducing transcription factors, TWIST1 and SLUG, in primary breast tumor tissue. These results suggest, that expression of EMT proteins in unselected tumor tissue is not surrogate marker of CTCs with either mesenchymal or epithelial features. Future studies will be need to identify expression of proteins in tumor tissue associated with presence of CTCs in the peripheral blood. These proteins could represent surrogate markers for biologically more aggressive disease and could represent potentially new therapeutic targets to inhibit metastatic process.

## Abbreviations

ANOVA: Analysis of variance
CD45+: CD45 positive
CTCs: Circulating tumor cells
ECM: Extracellular matrix
EMT: Epithelial-to-mesenchymal transition
EMT-TF: EMT-inducing transcription factors
ER: Estrogen receptor
HD: Healthy donors
H&E: Hematoxyllin and eosin
IRB: Institutional Review Board
IRS: ImmunoReactive Score
MBC: Metastatic breast cancer
PB: Peripheral blood
PBMC: Peripheral blood mononuclear cells
PR: Progesterone receptor
qRT-PCR: Quantitative real time polymerase chain reaction
SEM: Standard error of the mean
TF: Tissue factor

## References

[1] Cristofanilli M, Budd GT, Ellis MJ, Stopeck A, Matera J, Miller MC, Reuben JM, Doyle GV, Allard WJ, Terstappen LW, Hayes DF (2004). Circulating tumor cells, disease progression, and survival in metastatic breast cancer. N Engl J Med.

[2] Lucci A, Hall CS, Lodhi AK, Bhattacharyya A, Anderson AE, Xiao L, Bedrosian I, Kuerer HM, Krishnamurthy S (2012). Circulating tumour cells in non-metastatic breast cancer: a prospective study. Lancet Oncol.

[3] Zhang L, Riethdorf S, Wu G, Wang T, Yang K, Peng G, Liu J, Pantel K (2012). Meta-analysis of the prognostic value of circulating tumor cells in breast cancer. Clin Cancer Res.

[4] Zhao S, Liu Y, Zhang Q, Li H, Zhang M, Ma W, Zhao W, Wang J, Yang M (2011). The prognostic role of circulating tumor cells (CTCs) detected by RT-PCR in breast cancer: a metaanalysis of published literature. Breast Cancer Res Tr.

[5] Mego M, Mani SA, Cristofanilli M (2010). Molecular mechanisms of metastasis in breast cancer-clinical applications. Nat Rev Clin Oncol.

[6] Scheel C, Weinberg RA (2012). Cancer stem cells and epithelial-mesenchymal transition: concepts and molecular links. Semin Cancer Biol.

[7] Moustakas A, Heldin CH (2007). Signaling networks guiding epithelial-mesenchymal transitions during embryogenesis and cancer progression. Cancer Sci.

[8] Mani SA, Guo W, Liao MJ, Eaton EN, Ayyanan A, Zhou AY, Brooks M, Reinhard F, Zhang CC, Shipitsin M, Campbell LL, Polyak K, Brisken C, Yang J, Weinberg RA (2008). The epithelial-mesenchymal transition generates cells with properties of stem cells. Cell.

[9] Yang J, Weinberg RA (2008). Epithelial mesenchymal transition: at the crossroads of development and tumor metastasis. Dev Cell.

[10] Yang J, Mani SA, Donaher JL, Ramaswamy S, Itzykson RA, Come C, Savagner P, Gitelman I, Richardson A, Weinberg RA (2004). Twist, a master regulator of morphogenesis, plays an essential role in tumor metastasis. Cell.

[11] Wu ZQ, Li XY, Hu CY, Ford M, Kleer CG, Weiss SJ (2012). Canonical Wnt signaling regulates Slug activity and links epithelial-mesenchymal transition with epigenetic Breast Cancer 1, Early Onset (BRCA1) repression. Proc Natl Acad Sci U S A.

[12] Oka H, Shiozaki H, Kobayashi K, Inoue M, Tahara H, Kobayashi T, Takatsuka Y, Matsuyoshi N, Hirano S, Takeichi M (1993). Expression of E-cadherin cell adhesion molecules in human breast cancer tissues and its relationship to metastasis. Cancer Res.

[13] Aigner K, Dampier B, Descovich L, Mikula M, Sultan A, Schreiber M, Mikulits W, Brabletz T, Strand D, Obrist P, Sommergruber W, Schweifer N, Wernitznig A, Beug H, Foisner R, Eger A (2007). The transcription factor ZEB1 (deltaEF1) promotes tumour cell dedifferentiation by repressing master regulators of epithelial polarity. Oncogene.

[14] Anwar TE, Kleer CG (2013). Tissue-based identification of stem cells and epithelial-to-mesenchymal transition in breast cancer. Hum Pathol.

[15] van Nes JG, de Kruijf EM, Putter H, Faratian D, Munro A, Campbell F, Smit VT, Liefers GJ, Kuppen PJ, van de Velde CJ, Bartlett JM (2012). Co-expression of SNAIL and TWIST determines prognosis in estrogen receptor-positive early breast cancer patients. Breast Cancer Res Treat.

[16] Yuen HF, Zhang SD, Wong AS, McCrudden CM, Huang YH, Chan KY, El-Tanani M, Khoo US (2012). Regarding “Co-expression of SNAIL and TWIST determines prognosis in estrogen receptor-positive early breast cancer patients”. Breast Cancer Res Treat.

[17] Soini Y, Tuhkanen H, Sironen R, Virtanen I, Kataja V, Auvinen P, Mannermaa A, Kosma VM (2011). Transcription factors zeb1, twist and snai1 in breast carcinoma. BMC Cancer.

[18] Sarrió D, Rodriguez-Pinilla SM, Hardisson D, Cano A, Moreno-Bueno G, Palacios J (2008). Epithelial-mesenchymal transition in breast cancer relates to the basal-like phenotype. Cancer Res.

[19] Taube JH, Herschkowitz JI, Komurov K, Zhou AY, Gupta S, Yang J, Hartwell K, Onder TT, Gupta PB, Evans KW, Hollier BG, Ram PT, Lander ES, Rosen JM, Weinberg RA, Mani SA (2010). Core epithelial-to-mesenchymal transition interactome gene-expression signature is associated with claudin-low and metaplastic breast cancer subtypes. Proc Natl Acad Sci USA.

[20] Zhang Y, Toy KA, Kleer CG (2012). Metaplastic breast carcinomas are enriched in markers of tumor-initiating cells and epithelial to mesenchymal transition. Mod Pathol.

[21] Yu M, Bardia A, Wittner BS, Stott SL, Smas ME, Ting DT, Isakoff SJ, Ciciliano JC, Wells MN, Shah AM, Concannon KF, Donaldson MC, Sequist LV, Brachtel E, Sgroi D, Baselga J, Ramaswamy S, Toner M, Haber DA, Maheswaran S (2013). Circulating breast tumor cells exhibit dynamic changes in epithelial and mesenchymal composition. Science.

[22] Kasimir-Bauer S, Hoffmann O, Wallwiener D, Kimmig R, Fehm T (2012). Expression of stem cell and epithelial-mesenchymal transition markers in primary breast cancer patients with circulating tumor cells. Breast Cancer Res.

[23] Mego M, Mani SA, Lee BN, Li C, Evans KW, Cohen EN, Gao H, Jackson SA, GiordanoA HGN, Cristofanilli M, Lucci A, Reuben JM (2012). Expression of epithelial-mesenchymal transition-inducing transcription factors in primary breast cancer:The effect of neoadjuvant therapy. Int J Cancer.

[24] Cierna Z, Mego M, Janega P, Karaba M, Minarik G, Benca J, Sedlácková T, Cingelova S, Gronesova P, Manasova D, Pindak D, Sufliarsky J, Danihel L, Reuben JM, Mardiak J (2014). Matrix metalloproteinase 1 and circulating tumor cells in early breast cancer. BMC Cancer.

[25] Mego M, Karaba M, Minarik G, Benca J, Sedlácková T, Tothova L, Vlkova B, Cierna Z, Janega P, Luha J, Gronesova P, Pindak D, Fridrichova I, Celec P, Reuben JM, Cristofanilli M, Mardiak J (2015). Relationship between circulating tumor cells, blood coagulation, and urokinase-plasminogen-activator system in early breast cancer patients. Breast J.

[26] Xu Y, Hu B, Qin L, Zhao L, Wang Q, Wang Q, Xu Y, Jiang J (2014). SRC-1 and Twist1 expression positively correlates with a poor prognosis in human breast cancer. Int J Biol Sci..

[27] Molloy TJ, Roepman P, Naume B, van’t Veer LJ (2012). A prognostic gene expression profile that predicts circulating tumor cell presence in breast cancer patients. PLoS One.

